# Active abdominal compression-decompression cardiopulmonary resuscitation attenuates neuroinflammation associated with TLR4/NF-κB signaling in a rat model of cardiac arrest

**DOI:** 10.3892/etm.2026.13285

**Published:** 2026-09-04

**Authors:** Xiaopeng Liu, Xiaoqing Yang, Yifan Huang, Xinyuan Kong, Jiankai Gao, Hongyu Wang, Sisen Zhang

**Affiliations:** 1 Department of Emergency Medicine, The Fifth Clinical Medical College of Henan University of Chinese Medicine, Zhengzhou, Henan 450000, P.R. China; 2 Department of Emergency Medicine, People's Hospital of Henan University of Chinese Medicine/Zhengzhou People's Hospital, Zhengzhou, Henan 450000, P.R. China

**Keywords:** active abdominal compression-decompression cardiopulmonary resuscitation, cerebral ischemia-reperfusion injury, neuroinflammation, Toll-like receptor 4, nuclear factor-κB, cardiac arrest

## Abstract

Cardiac arrest (CA) induces cerebral ischemia-reperfusion injury, which is a major contributor to mortality and neurological dysfunction following resuscitation. Active abdominal compression-decompression cardiopulmonary resuscitation (AACD-CPR) has emerged as a potential alternative to standard cardiopulmonary resuscitation (STD-CPR). The present study aimed to investigate whether AACD-CPR attenuates neuroinflammation after CA/CPR and whether this effect is associated with changes in Toll-like receptor 4/nuclear factor-κB (TLR4/NF-κB)-related signaling. A rat model of CA was established, followed by resuscitation with either STD-CPR or AACD-CPR. Return of spontaneous circulation (ROSC), survival, NDS and neuronal injury were evaluated. The expression levels of TLR4, NF-κB and inflammation-related markers, including tumor necrosis factor-α, interleukin-1 β, glial fibrillary acidic protein (GFAP) and ionized calcium-binding adapter molecule 1 in brain tissue were assessed by western blotting and immunohistochemistry. Compared with STD-CPR, AACD-CPR markedly reduced resuscitation duration and improved early neurological outcomes. Although the ROSC rate and short-term survival rate were numerically higher in the AACD-CPR group than in the STD-CPR group, these differences did not reach statistical significance and therefore should not be interpreted as evidence of a definitive survival benefit. AACD-CPR was also associated with reduced neuronal injury, decreased expression of proinflammatory cytokines and glial activation markers and downregulation of TLR4/NF-κB-related proteins in brain tissue. AACD-CPR improved early neurological outcomes and attenuated neuroinflammatory responses in this rat model of CA/CPR. These effects were accompanied by suppression of TLR4/NF-κB-related signaling. However, as this was a preclinical small-animal study, the findings should be interpreted cautiously and not as evidence that AACD-CPR can clinically replace STD-CPR. Further studies in large-animal models and well-designed clinical investigations are required to validate its safety, feasibility and translational relevance.

## Introduction

Cardiac arrest (CA) is a major global public health challenge because of its sudden onset, narrow therapeutic window and high rates of mortality and disability (1). Cerebral ischemia-reperfusion injury (CIRI) refers to the exacerbation of ischemia-induced brain injury after the restoration of cerebral blood flow and oxygen supply. It is a major determinant of mortality and long-term neurological disability after CA and resuscitation (2,3), making prevention of CIRI-related brain injury critical for improving the prognosis of patients with CA. However, currently available therapeutic strategies remain unsatisfactory.

Standard cardiopulmonary resuscitation (STD-CPR), which is based primarily on chest compressions, has several limitations. Of patients undergoing chest compressions 30-80% may experience complications such as rib fractures, sternal fractures, or costochondral separation. These complications may not only injure organs such as the lungs, pleura and heart, but also affect the success of resuscitation (4). In addition, chest compressions may be contraindicated in patients with chest trauma, thoracic deformities, or other chest-related conditions. Active abdominal compression-decompression cardiopulmonary resuscitation (AACD-CPR) is an alternative resuscitation technique based on the principles of the ‘abdominal pump’ and ‘thoracic pump’ and was described in the expert consensus published by the China Active Abdominal Compression-Decompression Cardiopulmonary Resuscitation Collaborative Group (5). This approach alters intra-abdominal pressure through active abdominal compression and decompression and uses diaphragm-mediated abdominal pump effects to modify thoracic volume and intrathoracic-to-extrathoracic pressure gradients. By activating multiple pump mechanisms, including the thoracic pump, cardiac pump and pulmonary pump, AACD-CPR may simultaneously support artificial circulation and ventilation, thereby providing a novel approach to resuscitation. This technique may reduce secondary injuries, such as rib fractures, caused by conventional chest compressions and may provide an alternative resuscitation strategy for patients with contraindications to chest compression, including chest trauma or thoracic abnormalities (6). Animal studies have shown that, compared with STD-CPR, AACD-CPR can increase coronary perfusion pressure, improve ventilation efficiency and enhance resuscitation outcomes in experimental models of CA (7,8). Clinical studies have also suggested that AACD-CPR may be more effective than STD-CPR in improving neurological outcomes in patients with asphyxia-induced CA (9). Although AACD-CPR has shown potential for improving prognosis in clinical practice, the specific pathophysiological mechanisms underlying its beneficial effects remain to be elucidated.

Despite advances in resuscitation science, the overall survival rate of patients with CA remains low and a number of survivors experience sensorimotor and cognitive dysfunction, which is largely associated with CIRI induced by CA and CPR (1). CIRI involves a series of dynamic and complex pathophysiological processes, including excessive production of reactive oxygen species, increased expression of adhesion molecules, microglial activation and amplified inflammatory responses. These processes may further induce neuronal necrosis, apoptosis, blood-brain barrier disruption and cerebral edema (10). Neuroinflammation has increasingly been recognized as a central mechanism contributing to secondary brain injury after CA, linking CIRI, glial activation, oxidative stress and neurological dysfunction (11,12). When acute brain injury triggers an immune response, activated astrocytes and microglia release proinflammatory cytokines, such as interleukin-1 beta (IL-1β) and tumor necrosis factor-alpha (TNF-α), which promote the progression of neuroinflammation and subsequently induce cytotoxic effects (13).

Toll-like receptor 4 (TLR4) is an innate immune pattern-recognition receptor that recognizes various pathogen-associated molecular patterns and damage-associated molecular patterns (DAMPs), thereby initiating immune and inflammatory responses (14). Nuclear factor κB (NF-κB) is a key transcription factor involved in regulating the expression of multiple inflammatory mediators and immune-related genes (15). During CIRI, DAMPs released from damaged cells can be recognized by TLR4, leading to activation of the NF-κB signaling pathway. Activated NF-κB translocates to the nucleus and promotes the transcription of inflammatory mediators, including TNF-α and IL-1β, thereby amplifying the inflammatory response (16). Activation of the TLR4/NF-κB signaling pathway may also promote the expression of genes related to blood-brain barrier permeability, contributing to blood-brain barrier disruption and cerebral edema. Although the TLR4/NF-κB pathway has been implicated in CIRI by regulating inflammatory responses, its role in the effects of AACD-CPR remains unclear and warrants further investigation.

Based on the aforementioned evidence, it was hypothesized that AACD-CPR may mitigate CIRI after CA/CPR and that this protective effect may be associated with attenuation of TLR4/NF-κB-related neuroinflammatory signaling. The present study first compared resuscitation outcomes between STD-CPR and AACD-CPR in a rat model of CA and evaluated their effects on post-resuscitation neurological function. The present study then examined whether changes in neuroinflammation after AACD-CPR were accompanied by altered expression of TLR4/NF-κB pathway-related proteins. Given the absence of pathway-specific inhibitors or genetic manipulation, the present study was designed to provide preliminary associative evidence rather than definitive proof of pathway causality.

## Materials and methods

### Animals

A total of 40 male Sprague-Dawley rats, aged 13 weeks and weighing 300-400 g, were purchased from Beijing HFK Bioscience Co., Ltd. [Laboratory Animal Production License No. SCXK (Jing) 2019-0008]. All rats were housed under a 12-h light/dark cycle with *ad libitum* access to food and water. Prior to the experiment, the rats were acclimatized for 1 week under standard laboratory conditions, with a room temperature of 24.0˚C and a relative humidity of 50.0%. All animal experiments were approved by the Experimental Animal Ethics Committee of Henan University of Chinese Medicine, with the ethical approval number IACUC-202403026.

### Experimental groups and CA/CPR model

Rats were randomly assigned to three groups using a computer-generated randomization sequence: The STD-CPR group (n=15), in which rats received STD-CPR; the AACD-CPR group (n=15), in which rats received AACD-CPR; and the sham group (n=10), in which rats underwent tracheal intubation without CA or CPR.

All rats were fasted for 12 h before surgery. Anesthesia was induced by intraperitoneal injection of pentobarbital sodium at a dose of 45 mg/kg. The femoral artery was catheterized for arterial blood pressure monitoring and the femoral vein was catheterized to establish intravenous access. Tracheal intubation was then performed. Subsequently, the tracheal tube was clamped and the ventilator was turned off to induce asphyxia. After 7 min of untreated asphyxia, CPR was initiated by rapidly unclamping the tracheal tube and restarting the ventilator for assisted ventilation, while epinephrine was simultaneously administered through the femoral vein at a dose of 0.02 mg/kg.

In the STD-CPR group, cardiac compression was performed using a mechanical compression device for small animals (KW-XF small-animal cardiopulmonary resuscitation device; Nanjing Calvin Biotechnology Co., Ltd.). The compression site was located at the lower one-third of the sternum. Compressions were delivered at a rate of 200 compressions/min, with a compression depth equal to one-third of the anteroposterior diameter of the thorax. In the AACD-CPR group, AACD-CPR was performed using an abdominal compression-decompression CPR device (ES-3910 vacuum pump mini 24V suction pump; Shenzhen Pincheng Motor Co, Ltd.). The connection port of the suction cup was placed on the upper abdomen of each rat and the operator continuously alternated vertical downward compression and upward decompression at a frequency of 200 cycles/min. The compression-decompression amplitude was equal to half of the anteroposterior diameter of the thorax (17). Return of spontaneous circulation (ROSC) was defined as the presence of a sinus rhythm with mean arterial pressure (MAP) ≥60 mmHg maintained for at least 10 min. Mechanical ventilation was withdrawn 1 h after ROSC when blood pressure and spontaneous respiration were stable.

### Evaluation of survival rate, CPR duration and neurological deficits

CPR duration was defined as the time from the initiation of resuscitation to ROSC. Survival rates in each group were calculated on postoperative days 1-3. From postoperative days 1-3, neurological function was assessed daily by an investigator blinded to group allocation using the neurological deficit score (NDS) (18). The NDS evaluates consciousness, reflexes, motor function, sensation and balance responses, with scores ranging from 0-80. A score of 0 indicates brain death, whereas a score of 80 indicates normal neurological function. Higher scores indicate less severe neurological damage, whereas lower scores indicate more severe neurological impairment (19).

### Tissue preparation

On postoperative day 3, at the predetermined experimental endpoint, the rats were sacrificed by intraperitoneal injection of an overdose of sodium pentobarbital at a dose of 100 mg/kg body weight. The animals were continuously monitored until irreversible loss of consciousness, cessation of spontaneous respiration and absence of heartbeat were confirmed before tissue collection. The rats were then perfused through the aorta with cold physiological saline followed by 4% paraformaldehyde pre-cooled to 4˚C. The brain was immediately removed after decapitation, fixed in 4% paraformaldehyde at 4˚C for 24 h. Following fixation, the brain tissues were dehydrated through a graded ethanol series, cleared in xylene, infiltrated with molten paraffin and embedded in paraffin blocks. Subsequently, 3-µm-thick paraffin sections were prepared for hematoxylin and eosin (H&E) staining and immunohistochemistry (IHC) and 5-µm-thick paraffin sections were prepared for Nissl staining.

### H&E and Nissl staining

Brain tissue sections were deparaffinized in xylene and rehydrated through a graded ethanol series consisting of 100, 95, 75 and 50% ethanol. The sections were then subjected to H&E staining, air-dried, mounted with coverslips and observed under an optical microscope (Olympus Corporation).

For Nissl staining, paraffin-embedded sections were deparaffinized in xylene and rehydrated through a graded ethanol series consisting of 100, 95, 75 and 50% ethanol. The sections were stained with toluidine blue solution in a sealed humid chamber at 60˚C for 30 min and then counterstained with 0.5% eosin for 3 sec. Images were captured under an optical microscope. Nissl bodies were counted in five randomly selected fields from each group using ImageJ 1.46 software (National Institutes of Health).

### IHC

Immunohistochemistry was performed to evaluate the expression of TNF-α and IL-1β in brain tissue sections. Following deparaffinization and rehydration, heat-induced antigen retrieval was performed in citrate buffer. Endogenous peroxidase activity was quenched by incubating the sections with 3% hydrogen peroxide at room temperature for 25 min. After washing with PBS, the sections were blocked with 3% bovine serum albumin (BSA; Beijing Solarbio Science & Technology Co., Ltd.) at room temperature for 30 min. The blocking solution was discarded without washing, and the sections were incubated overnight at 4˚C with primary antibodies against TNF-α (cat. no. ab307164; 1:1,000; Abcam) and IL-1β (cat. no. ab283818; 1:100; Abcam). Following washing, the sections were incubated with HRP-conjugated goat anti-rabbit IgG secondary antibody (cat. no. GB23303; 1:200; Beijing Solarbio Science & Technology Co., Ltd.) at room temperature for 50 min. Immunoreactivity was visualized using 3,3'-diaminobenzidine chromogen (DAB; cat. no. G1211; Beijing Solarbio Science & Technology Co., Ltd.), and the nuclei were counterstained with hematoxylin at room temperature for 3 min. The sections were examined using a bright-field light microscope at x400 magnification and calibrated scale bars of 50 µm were included in the representative images. Histological image acquisition and analysis were performed by investigators blinded to the experimental group assignments.

### Western blot analysis

Following sacrifice, rat brains were immediately removed, with three rats included in each group. The brain tissues were rinsed with ice-cold physiological saline and rapidly stored at -80˚C until western blot analysis. Hippocampal tissue samples, with 50 mg used for each sample, were lysed and homogenized on ice. The extracted proteins were centrifuged at 12,000 x g for 15 min at 4˚C and the supernatant was collected. Total protein concentration in the lysate was measured using a BCA protein assay kit (cat. no. PK10026; Proteintech Group, Inc.).

Equal amounts of protein (30 µg per lane) were separated using 12% SDS-PAGE gels and subsequently transferred onto polyvinylidene fluoride membranes (cat. no. IPVH00010; 0.2-µm pore size; MilliporeSigma) and blocked with 5% skimmed milk at room temperature for 2 h. The membranes were then incubated overnight at 4˚C with the corresponding primary antibodies. The primary antibodies used were as follows: anti-TLR4 (19811-1-AP; 1:2,000; Proteintech Group, Inc.), anti-NF-κB (ab32360; 1:5,000; Abcam), anti-TNF-α (ab307104; 1:1,000; Abcam), anti-IL-1β (ab283818; 1:1,000; Abcam), anti-ionized calcium-binding adapter molecule 1 (Iba1; ab178846; 1:1,000; Abcam), anti-GFAP (ab7260; 1:10,000; Abcam), anti-β-actin (20536-1-AP; 1:4,000; Proteintech Group, Inc.) and anti-GAPDH (AF7021-50; 1:3,000; Affinity).

After washing with TBST containing 0.1% Tween-20,the membranes were incubated with HRP-conjugated secondary antibodies (cat. no. SA00001-2; 1:5,000; Proteintech Group, Inc.) at room temperature for 2 h. Target proteins were detected using enhanced chemiluminescence and images were captured using a chemiluminescence detection system (GeneGenius; Syngene International). The optical density of protein bands was quantified using ImageJ 1.46 software (National Institutes of Health) and normalized to β-actin or GAPDH. Western blot densitometric semi-quantification was performed by investigators blinded to group allocation.

### Statistical analysis

Statistical analysis was performed using SPSS 27.0 (IBM Corp.) and GraphPad Prism 9.5.0 (Dotmatics). Bar graphs were generated using GraphPad Prism 9.5.0. Count data are expressed as numbers and percentages and comparisons between groups were performed using the χ² test or Fisher's exact test, as appropriate. Continuous data are presented as the mean ± standard deviation (SD). Comparisons between two groups were performed using Student's t-test, whereas comparisons among multiple groups were performed using one-way analysis of variance followed by Tukey's post hoc multiple-comparisons test. P<0.05 was considered to indicate a statistically significant difference.

## Results

### Effects of AACD-CPR on survival rate, CPR duration and NDS following CA

As shown in Table I, the ROSC rate and short-term survival rate were numerically higher in the AACD-CPR group compared with the STD-CPR group; however, these differences were not statistically significant (P>0.05). Therefore, these findings should not be interpreted as evidence of a definitive survival benefit of AACD-CPR in the present animal model. By contrast, AACD-CPR markedly shortened CPR duration and improved early NDS compared with STD-CPR (P<0.05), suggesting improved resuscitation efficiency and early neurological recovery.

### AACD-CPR alleviates morphological damage to neurons in the brain tissue of CA/CPR rats

**Figure 1:**
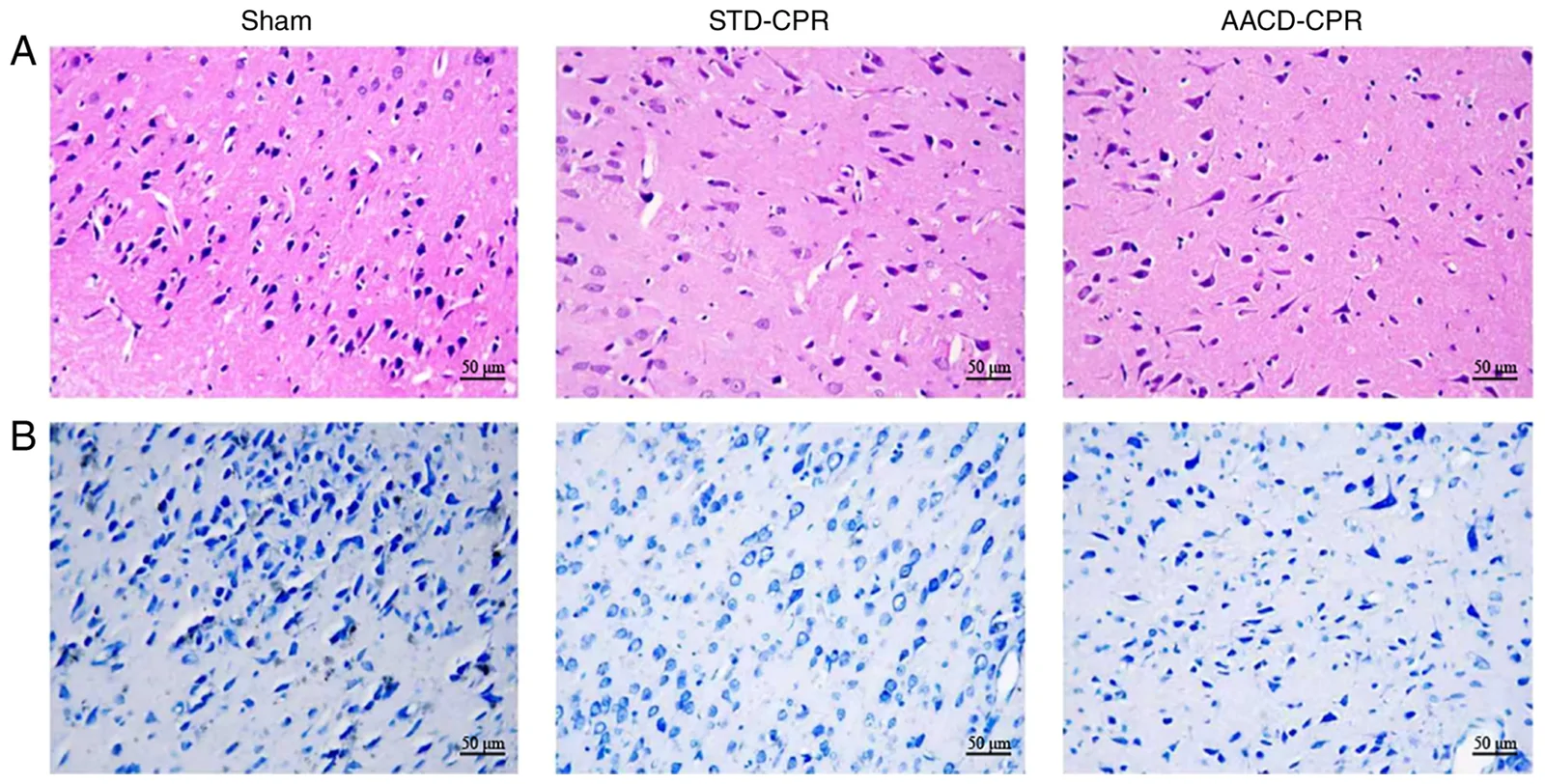
H&E and Nissl staining of brain tissue 72 h after ROSC following CA/CPR or at the corresponding time point after sham surgery. (A) H&E staining of brain tissue showing neuronal morphology, including normal neurons and neurons exhibiting vacuolation or cell death, in the Sham, STD-CPR and AACD-CPR groups. (B) Nissl staining of brain tissue showing viable neurons in the Sham, STD-CPR and AACD-CPR groups. Representative images were captured at x400 magnification (scale bar, 50 µm). H&E, hematoxylin and eosin; ROSC, return of spontaneous circulation; CA, cardiac arrest; CPR, cardiopulmonary resuscitation; STD-CPR, standard cardiopulmonary resuscitation; AACD-CPR, active abdominal compression-decompression cardiopulmonary resuscitation.

To compare the effects of STD-CPR and AACD-CPR on CIRI, neuronal injury was evaluated by assessing morphological changes in brain tissue. As shown in Fig. 1, H&E staining in the sham group showed normal neuronal morphology, abundant neuronal cells, relatively uniform cellular arrangement, organized tissue fibers and no obvious cellular vacuolization or necrosis. Nissl staining revealed abundant Nissl bodies in the sham group. By contrast, rats subjected to CA/CPR exhibited marked neuronal damage, including necrotic cells, nuclear pyknosis, cellular edema, vacuolization and a substantial reduction in Nissl bodies. Compared with the STD-CPR group, the AACD-CPR group showed improved neuronal morphology on H&E staining, with only mild swelling and nuclear pyknosis observed in a small number of neurons. In addition, reduced neuronal death and an increased number of Nissl bodies were observed in the AACD-CPR group.

### AACD-CPR is associated with downregulation of TLR4 and NF-κB in the hippocampus of CA/CPR rats

**Figure 2:**
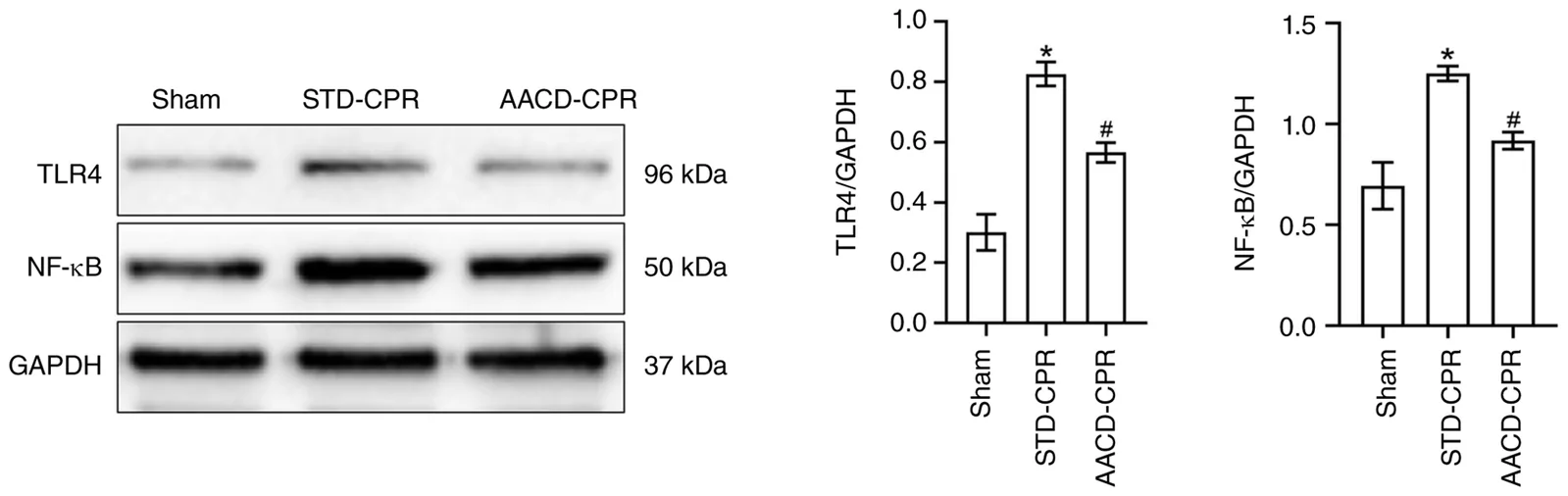
Western blot analysis of TLR4 and NF-κB expression. ^*^P<0.05 vs. sham, ^#^P<0.05 vs. STD-CPR.TLR4, Toll-like receptor 4; NF-κB, nuclear factor-κB.STD-CPR, standard cardiopulmonary resuscitation; AACD-CPR, active abdominal compression-decompression cardiopulmonary resuscitation.

The expression levels of TLR4 and NF-κB in the hippocampus were semi-quantified by western blot analysis. Compared with the sham group, the STD-CPR group showed markedly increased expression of TLR4 and NF-κB (P<0.05). By contrast, TLR4 and NF-κB expression levels were significantly lower in the AACD-CPR group than in the STD-CPR group (P<0.05; Fig. 2). These findings suggested that AACD-CPR was associated with suppression of TLR4/NF-κB-related signaling after CA/CPR. However, these results do not establish whether this pathway is causally required for the neuroprotective effects of AACD-CPR.

### AACD-CPR downregulates proinflammatory cytokines and glial activation markers in the hippocampus of CA/CPR rats

**Figure 3:**
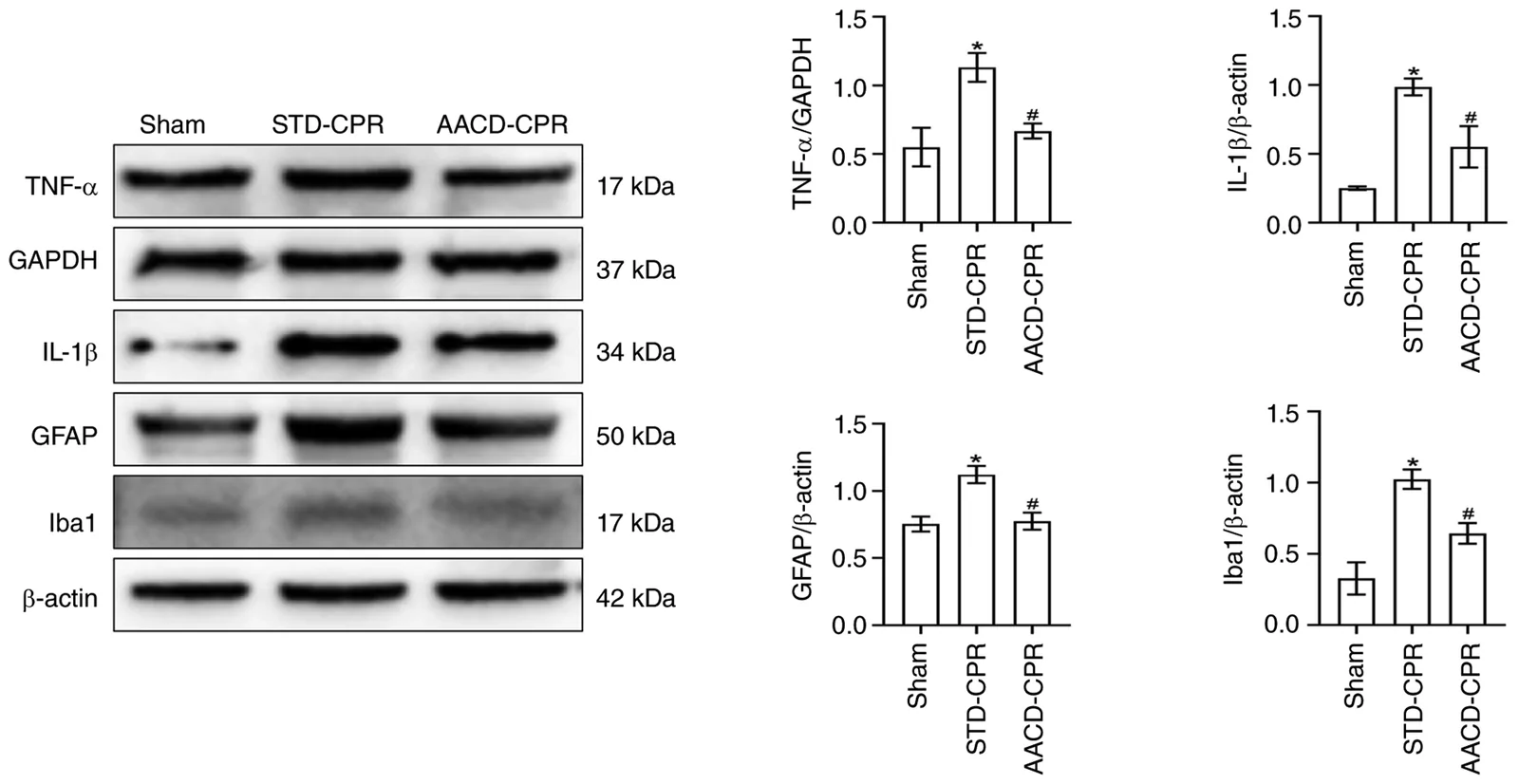
Western blot analysis of TNF-α, IL-1β, GFAP and Iba1 expression. ^*^P<0.05 vs. sham, ^#^P<0.05 vs. STD-CPR. TNF-α, tumor necrosis factor-alpha; IL-1β, interleukin-1 beta; GFAP, glial fibrillary acidic protein; Iba1, ionized calcium-binding adapter molecule 1.STD-CPR, standard cardiopulmonary resuscitation; AACD-CPR, active abdominal compression-decompression cardiopulmonary resuscitation.

**Figure 4:**
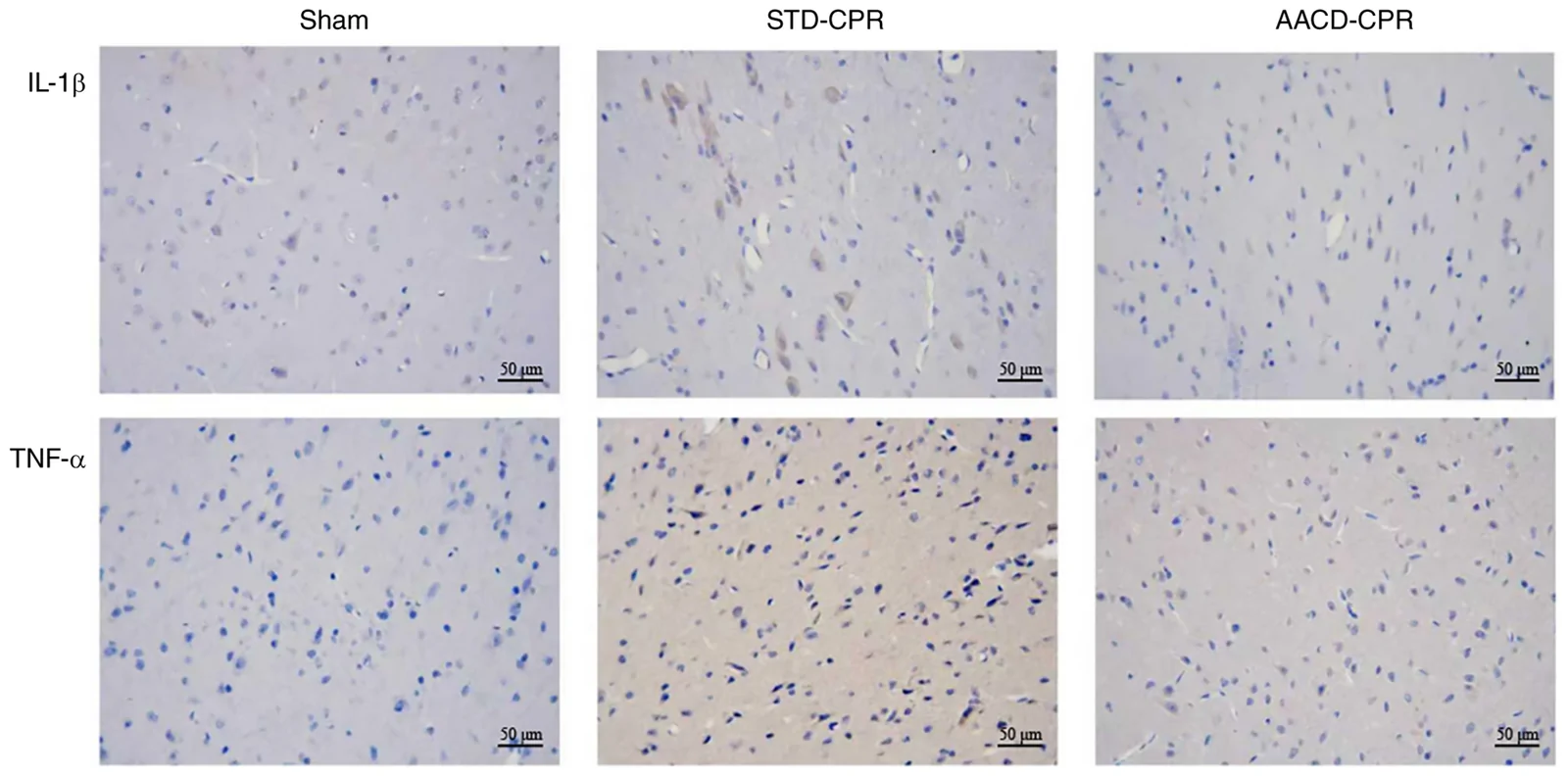
Immunohistochemical staining of IL-1β and TNF-α in brain tissue from the sham, STD-CPR and AACD-CPR groups. Representative images were captured at x400 magnification (scale bar, 50 µm). IL-1β, interleukin-1 beta; TNF-α, tumor necrosis factor-alpha; STD-CPR, standard cardiopulmonary resuscitation; AACD-CPR, active abdominal compression-decompression cardiopulmonary resuscitation.

Western blotting and immunohistochemistry were performed to assess the expression levels of the proinflammatory cytokines TNF-α and IL-1β, as well as the glial activation markers GFAP and Iba1, in the hippocampus of rats after CA/CPR. As shown in Figs. 3 and 4, the expression levels of TNF-α, IL-1β, GFAP and Iba1 were markedly increased in the STD-CPR group compared with the sham group (P<0.05). By contrast, AACD-CPR markedly reduced the expression levels of TNF-α, IL-1β, GFAP and Iba1 compared with STD-CPR (P<0.05). These results indicated that AACD-CPR attenuated inflammatory cytokine expression and glial activation in the hippocampus after CA/CPR.

## Discussion

In the present study, AACD-CPR was associated with a markedly shorter time to ROSC than STD-CPR in a rat model of CA. In addition, AACD-CPR improved early NDS, alleviated histopathological neuronal injury and reduced the expression of proinflammatory cytokines and glial activation markers in brain tissue. These effects were accompanied by downregulation of TLR4 and NF-κB expression. Together, these findings suggest that AACD-CPR may improve early resuscitation efficiency and attenuate post-resuscitation neuroinflammatory responses in this experimental model. However, given the small-animal design, limited sample size, short observation period and lack of pathway-specific intervention, these findings should be interpreted as preliminary and associative rather than definitive evidence of mechanistic causality or clinical superiority.

The time to ROSC was markedly shorter in the AACD-CPR group compared with the STD-CPR group (66.4±9.56 vs. 91.8±7.35 sec), suggesting that AACD-CPR may improve the efficiency of early resuscitation in this rat model. From a physiological perspective, AACD-CPR may enhance circulatory support by cyclically altering intra-abdominal and intrathoracic pressures. During the decompression phase, decreased intra-abdominal pressure may facilitate downward movement of the diaphragm, reduce intrathoracic pressure and promote venous return to the heart. During the compression phase, increased intra-abdominal pressure may elevate the diaphragm and increase intrathoracic pressure, thereby contributing to forward blood flow. These pressure-mediated pump effects may partly explain the shorter resuscitation time observed in the AACD-CPR group.

However, the clinical significance of this finding should be interpreted cautiously. Although the ROSC rate was numerically higher in the AACD-CPR group than in the STD-CPR group (73.3 vs. 66.7%), the difference was not statistically significant. Similarly, the short-term survival rate was slightly higher in the AACD-CPR group, but this difference also did not reach statistical significance. Therefore, the present results did not demonstrate a clear advantage of AACD-CPR over STD-CPR in terms of ROSC rate or 72-h cumulative survival. Rather, these findings suggested that AACD-CPR may achieve comparable short-term resuscitation outcomes to STD-CPR while reducing the time required to achieve ROSC in this experimental setting.

In clinical practice, STD-CPR remains the standard and widely validated resuscitation method. AACD-CPR may have potential value as an alternative or adjunctive approach in selected situations where conventional chest compressions are difficult or may increase the risk of injury, such as severe chest wall trauma, thoracic deformity, or multiple rib fractures. Nevertheless, this possibility remains speculative and requires further validation. As the present study was conducted in a small-animal model, its findings cannot be directly extrapolated to clinical populations. Additional large-animal studies and well-designed clinical investigations are required to determine whether AACD-CPR can improve clinically meaningful outcomes, including sustained ROSC, long-term survival and neurological recovery. Moreover, the safety, feasibility, operator training requirements and device-related factors of AACD-CPR should be carefully evaluated before clinical application.

Neurological recovery after CA is a major determinant of prognosis after resuscitation. In the present study, neurological function was evaluated using NDS within 72 h after ROSC. Rats in both the STD-CPR and AACD-CPR groups exhibited neurological impairment after CA/CPR, followed by gradual recovery over time. No significant difference in NDS was observed between the two groups at 24 or 48 h following ROSC. However, at 72 h after ROSC, neurological function was markedly improved in the AACD-CPR group compared with the STD-CPR group. These findings suggested that AACD-CPR may provide a potential early neurological benefit after CA/CPR.

CIRI is considered a major contributor to neurological dysfunction following CA and resuscitation (20,21). AACD-CPR may attenuate IRI brain injury by improving the restoration of systemic and cerebral circulation during resuscitation. Compared with STD-CPR, AACD-CPR may help maintain cerebral perfusion and oxygen delivery, thereby reducing neuronal damage and cell death (22). In addition, AACD-CPR may exert neuroprotective effects through other mechanisms, including modulation of neuroinflammatory responses and oxidative stress (23). Nevertheless, the current neurological assessment was limited to the first 72 h following ROSC. Although improved NDS suggests beneficial early neurological recovery, it remains unclear whether this improvement persists over longer observation periods. Neurological recovery after CA may continue for weeks to months and may involve complex processes, including neuronal remodeling, glial activation, delayed neuroinflammation and synaptic repair. Therefore, the present findings should be interpreted as evidence of early, rather than long-term, neurological protection.

Histopathological analysis further supported the potential neuroprotective effect of AACD-CPR. H&E staining showed marked neuronal damage after CA/CPR, including neuronal necrosis, nuclear pyknosis, cellular edema and vacuolization. Nissl staining also revealed a substantial reduction in Nissl bodies, indicating neuronal injury. Compared with STD-CPR, AACD-CPR alleviated these morphological abnormalities and increased the number of Nissl bodies. These findings were consistent with the observed improvement in early neurological function and suggest that AACD-CPR may reduce neuronal structural injury after resuscitation.

Neuroinflammation plays a critical role in secondary brain injury after CA. Following cerebral IRI, damaged cells release damage-associated molecular patterns, which can activate innate immune signaling pathways and amplify inflammatory responses.

In the present study, the expression levels of TLR4 and NF-κB were markedly increased in brain tissue after CA/CPR, whereas AACD-CPR markedly reduced their expression compared with STD-CPR. TLR4 is a pattern-recognition receptor involved in innate immune and inflammatory responses and its activation can initiate downstream signaling cascades that promote cytokine release and tissue injury. NF-κB is a key transcription factor that regulates the expression of multiple inflammatory genes, including TNF-α and IL-1β (24-26). A recent study of CIRI has also shown that activation of the HMGB1/TLR4/NF-κB axis contributes to microglial activation, cytokine release and neuronal injury (27).

Consistent with these mechanisms, rats in the STD-CPR group showed markedly increased expression of TNF-α, IL-1β, GFAP and Iba1, whereas these markers were reduced following AACD-CPR. TNF-α and IL-1β are major proinflammatory cytokines involved in post-ischemic neuroinflammation, whereas GFAP and Iba1 are commonly used markers of astrocyte and microglial activation, respectively. The reduction of these markers in the AACD-CPR group suggested that AACD-CPR attenuated inflammatory cytokine production and glial activation after CA/CPR. These findings indicated that the early neurological improvement observed after AACD-CPR was accompanied by suppression of TLR4/NF-κB-associated neuroinflammatory activation.

However, the mechanistic interpretation of these findings requires caution. As the present study did not include TLR4 or NF-κB inhibitor-treated groups, pathway-specific antagonists, or genetic knockout models, a direct causal relationship between suppression of the TLR4/NF-κB pathway and the neuroprotective effects of AACD-CPR cannot be established. Therefore, the results provided evidence of an association between AACD-CPR and reduced TLR4/NF-κB-related signaling rather than definitive proof that this pathway mediates the observed protection.

An alternative explanation is that the reduction in TLR4/NF-κB signaling may be secondary to improved physiological recovery during resuscitation. CIRI is highly dependent on blood flow restoration and tissue oxygenation. Improved hemodynamic performance during AACD-CPR may enhance cerebral perfusion and oxygen delivery, thereby reducing ischemic burden and subsequently attenuating neuroinflammatory activation. Consequently, the observed anti-inflammatory effects may represent downstream consequences of improved cerebral perfusion rather than direct modulation of inflammatory signaling pathways.

In addition, the inflammatory markers evaluated in the present study primarily reflected acute glial activation and cytokine production. Neuroinflammation following CA is considerably more complex and involves diverse microglial phenotypes, astrocytic responses, inflammasome activation, chemokine signaling, oxidative stress pathways, blood-brain barrier disruption and interactions between immune and neuronal cells. Therefore, the current findings should be regarded as a preliminary characterization of neuroinflammatory changes rather than a comprehensive assessment of the inflammatory landscape after CA/CPR.

The novelty of the present study lies in its multidimensional evaluation of AACD-CPR, including resuscitation efficiency, early neurological function, histopathological injury, inflammatory markers and TLR4/NF-κB-associated signaling. Nevertheless, several limitations should be acknowledged. First, no pharmacological inhibition or genetic manipulation was performed to establish the causal role of the TLR4/NF-κB pathway. Second, neurological and inflammatory outcomes were assessed only during the acute phase, up to 72 h following ROSC, precluding conclusions regarding long-term neuroprotection or chronic neuroinflammation. Third, cerebral blood flow, cerebral perfusion pressure and brain tissue oxygenation were not directly measured, making it difficult to determine the relative contribution of hemodynamic improvement vs. inflammatory modulation. Fourth, although outcome assessment was performed in a blinded manner, operator blinding during resuscitation was not feasible because of the substantial procedural differences between AACD-CPR and STD-CPR; therefore, some degree of performance bias cannot be excluded. Finally, the present study was conducted in a small-animal model with a limited sample size and further validation in large-animal models and clinical populations is required.

Future studies should incorporate cerebral blood flow monitoring, cerebral perfusion pressure measurements, brain tissue oxygenation assessment, serial evaluation of inflammatory mediators and pathway-specific pharmacological or genetic interventions. Such studies will help clarify the mechanisms underlying AACD-CPR-mediated neuroprotection and determine the relative contributions of hemodynamic improvement and inflammatory pathway modulation. Long-term behavioral assessments and survival analyses are also needed to evaluate whether the early neurological benefits observed in the present study translate into sustained functional recovery.

## Figures and Tables

**Table I:** ROSC rate, CPR duration, survival rate and neurological deficit score.

Group	Sham (n=10)	STD-CPR (n=15)	AACD-CPR (n=15)
ROSC	NA	10/15 (67%)	11/15 (73%)
Day 1 survival rate	10/10 (100%)	8/15 (53%)	10/15 (67%)
Day 2 survival rate	10/10 (100%)	8/15 (53%)	10/15 (67%)
Day 3 survival rate	10/10 (100%)	8/15 (53%)	10/15 (67%)
Number	10	8	10
CPR duration(s)	-	91.8±7.35	66.4±9.56^b^
Day 1 NDS	80.00±0.00	59.8±1.83^a^	60.4±1.9^a^
Day 2 NDS	80.00±0.00	63.0±1.41^a^	64.2±2.0^a^
Day 3 NDS	80.00±0.00	67.5±1.60^a^	72.8±1.4^a,b^

Comparison of ROSC, survival rates on postoperative days 1-3, CPR duration and NDS at various time points among the three groups. Resuscitation and survival rates are expressed as percentages. CPR duration and NDS are presented as mean ± SD.

^a^P<0.05 vs. sham group;

^b^P<0.05 vs. STD-CPR group. ROSC, return of spontaneous circulation; CPR, cardiopulmonary resuscitation; CA, cardiac arrest; Sham, sham (surgery) group; STD-CPR, standard cardiopulmonary resuscitation; AACD-CPR, active abdominal compression-decompression cardiopulmonary resuscitation; CPR duration, cardiopulmonary resuscitation duration; NDS, neurological deficit score.

## Data Availability

The data generated in the present study are included in the figures and/or tables of this article.
